# A case of insulin allergy in a girl with type 1 diabetes

**DOI:** 10.1186/1687-9856-2013-S1-P9

**Published:** 2013-10-03

**Authors:** Annang Giri Moelyo, Nova Ardyanto

**Affiliations:** 1 Child Health Department, Faculty of Medicine, Sebelas Maret University Moewardi Hospital, Surakarta, Indonesia

## 

Insulin is an important treatment for type 1 diabetes. Allergy caused by insulin administration is very rare. Several studies have reported some cases of insulin allergy caused by insulin administration. This is the first case of insulin allergy in our hospital.

A 12 years old girl came to outpatient ward with oedema. She had oedema in palpebra, lower extremities and ascites. There were no dyspneu, abdominal pain, and urination complaint. She has been 4 years treatment of type 1 diabetes. In last visit, she was hospitalised because of worst glycemic results (frequent episode of hypoglycemia, hyperglycemia, and HbA1c 16.4%). She was changed from conventional regimen (twice daily) to multiple daily regimen, with the use of twice daily mix insulin (intermediate and rapid) plus rapid insulin (aspart insulin), 3 days before the oedema. There were no involvement of hepatic, cardiac and renal disorder. Cetirizine was given, and the oedema disappeared. We changed back to twice daily regimen for several days, and then try to multiple daily regimen. While she uses multiple daily regimen, oedema occurred again. After discontinuing rapid insulin (aspart insulin), she never had this allergy. She uses a mix-split regimen now. Insulin allergy in this case could be caused by the insulin or non-medical ingredients. As a conclusion, we must concern to insulin allergy although a very rare case.

